# Growth Hormone Research Society perspective on the development of long-acting growth hormone preparations

**DOI:** 10.1530/EJE-16-0111

**Published:** 2016-06

**Authors:** Jens Sandahl Christiansen, Philippe F Backeljauw, Martin Bidlingmaier, Beverly M K Biller, Margaret C S Boguszewski, Felipe F Casanueva, Philippe Chanson, Pierre Chatelain, Catherine S Choong, David R Clemmons, Laurie E Cohen, Pinchas Cohen, Jan Frystyk, Adda Grimberg, Yukihiro Hasegawa, Morey W Haymond, Ken Ho, Andrew R Hoffman, Jeff M P Holly, Reiko Horikawa, Charlotte Höybye, Jens Otto L Jorgensen, Gudmundur Johannsson, Anders Juul, Laurence Katznelson, John J Kopchick, K O Lee, Kuk-Wha Lee, Xiaoping Luo, Shlomo Melmed, Bradley S Miller, Madhusmita Misra, Vera Popovic, Ron G Rosenfeld, Judith Ross, Richard J Ross, Paul Saenger, Christian J Strasburger, Michael O Thorner, Haim Werner, Kevin Yuen

**Affiliations:** 1 Aarhus UniversityAarhus, Denmark; 2 Cincinnati Children’s HospitalCincinnati, Ohio, USA; 3 Klinikum der Universität MünchenMunich, Germany; 4 Massachusetts General HospitalBoston, Massachusetts, USA; 5 Federal University of ParanaCuritiba, Brazil; 6 Santiago de Compostela UniversitySantiago de Compostela, Spain; 7 Université Paris SaclayLe Kremlin-BicϪtre, France; 8 University Claude BernardLyon, France; 9 University of Western AustraliaSubiaco, Western Australia, Australia; 10 University of North CarolinaChapel Hill, North Carolina, USA; 11 Boston Children’s HospitalBoston, Massachusetts, USA; 12 University of Southern CaliforniaLos Angeles, California, USA; 13 Children’s Hospital of PhiladelphiaPhiladelphia, Pennsylvania, USA; 14 Tokyo Metropolitan Children’s Medical CenterTokyo, Japan; 15 Baylor College of MedicineHouston, Texas, USA; 16 University of QueenslandBrisbane, Queensland, Australia; 17 VA Palo Alto Health Care System and Stanford UniversityPalo Alto, California, USA; 18 University of BristolBristol, UK; 19 National Center for Child Health and DevelopmentTokyo, Japan; 20 Karolinska University HospitalStockholm, Sweden; 21 University of GothenburgGothenburg, Sweden; 22 RigshospitaletUniversity of Copenhagen, Copenhagen, Denmark; 23 Stanford UniversityStanford, California, USA; 24 Ohio UniversityAthens, Ohio, USA; 25 National University of SingaporeSingapore, Singapore; 26 UCLA School of MedicineLos Angeles, California, USA; 27 Tongji HospitalWuhan, China; 28 Cedars-Sinai Medical CenterLos Angeles, California, USA; 29 Masonic Children’s HospitalMinneapolis, Minnesota, USA; 30 University of BelgradeBelgrade, Serbia; 31 Stat5 ConsultingLos Altos, California, USA; 32 Jefferson Medical CollegePhiladelphia, Pennsylvania, USA; 33 University of SheffieldSheffield, UK; 34 SUNYStony Brook, New York, USA; 35 Charite-UniversitätsmedizinBerlin, Germany; 36 University of VirginiaCharlottesville, Virginia, USA; 37 Tel Aviv UniversityTel Aviv, Israel; 38 Swedish Neuroscience InstituteSeattle, Washington, USA

## Abstract

**Objective:**

The Growth Hormone (GH) Research Society (GRS) convened a workshop to address important issues regarding trial design, efficacy, and safety of long-acting growth hormone preparations (LAGH).

**Participants:**

A closed meeting of 55 international scientists with expertise in GH, including pediatric and adult endocrinologists, basic scientists, regulatory scientists, and participants from the pharmaceutical industry.

**Evidence:**

Current literature was reviewed for gaps in knowledge. Expert opinion was used to suggest studies required to address potential safety and efficacy issues.

**Consensus process:**

Following plenary presentations summarizing the literature, breakout groups discussed questions framed by the planning committee. Attendees reconvened after each breakout session to share group reports. A writing team compiled the breakout session reports into a draft document that was discussed and revised in an open forum on the concluding day. This was edited further and then circulated to attendees from academic institutions for review after the meeting. Participants from pharmaceutical companies did not participate in the planning, writing, or in the discussions and text revision on the final day of the workshop. Scientists from industry and regulatory agencies reviewed the manuscript to identify any factual errors.

**Conclusions:**

LAGH compounds may represent an advance over daily GH injections because of increased convenience and differing phamacodynamic properties, providing the potential for improved adherence and outcomes. Better methods to assess adherence must be developed and validated. Long-term surveillance registries that include assessment of efficacy, cost-benefit, disease burden, quality of life, and safety are essential for understanding the impact of sustained exposure to LAGH preparations.

## Background

The long-term safety and efficacy of daily recombinant human growth hormone (rhGH) have been well documented since its approval by the United States Food and Drug Administration (FDA) in 1985 and by the European Medicines Agency (EMA) in 1987. However, daily GH injections can be burdensome for some patients and caregivers. Furthermore, variability in clinical outcomes is well recognized in clinical practice, and may reflect differences in adherence to prescribed therapy (1). Published data and clinical experience indicate that nonadherence to daily rhGH therapy increases over time (2, 3, 4, 5, 6, 7, 8) and impairs therapeutic responses. Many life circumstances can interfere with adherence to daily injections, thus compromising therapeutic outcomes. By decreasing injection frequency, long-acting GH (LAGH) formulations may improve adherence and thereby potentially maximize clinical efficacy. Consequently, there are currently several LAGH preparations in various stages of preclinical development, with some already approved for clinical use (Table 1).

**Table 1:** Long-acting GH preparations.

	**Company**	**Product**	**Modification to the GH molecule**	**Frequency of administration**	**Current status**
**Depot chemical**			**Depot formulations**		
	Genentech	Nutropin Depot	Encapsulated in biocompatible, biodegradable, polylactide-coglycolide polymer microspheres	14 days	Removed from the market
	LG Life Sciences, Ltd.	LB3002	Microparticles containing GH incorporated into sodium hyaluronate and dispersed in an oil base of medium-chain triglycerides	7 days	Marketed in Korea for childhood GHD Approved in Europe, but not marketed in the EU
	Altus Pharmaceuticals	ALTU-238	Protein crystallization technology	7 days	No longer being developed
	Critical Pharmaceuticals	CP016	Supercritical carbon dioxide, formed when CO_2_ exceeds its thermodynamic critical point, used to create the depot	2 weeks (planned)	Preclinical studies
**PEGylated formulations**			**PEGylation**		
	Ambrx	ARX201	30-kDa PEG added to unnatural amino acid incorporated into GH	7 days	No longer being developed
	Novo Nordisk A/S	NNCI126-0083	43-kDa PEG residue attached to glutamine 141	7 days	No longer being developed
	Pfizer	PEG-GH PHA-794428	Branched 40-kDa PEG on N terminus of GH	7 days	No longer being developed
	Bolder BioTechnology	BBT-031	Site-specific PEGylated GH analog	7 days (planned)	Preclinical studies
	GeneScience Pharmaceuticals Co., Ltd.	Jintrolong	40-kDa PEG attached to GH	7 days	Marketed in China for childhood GHD
**Prodrug formulations**			**Mechanism of conversion to active drug**		
	Ascendis Pharma A/S	TransCon PEG GH (ACP-001)	hGH transiently bound to a PEG carrier molecule via a self-cleaving linker. The released GH is unmodified	7 days	Phase II studies in children and adults
**Noncovalent albumin binding GH compound(s)**			**Albumin binding**		
	Novo Nordisk	NNbib195-0092	Single-point mutation in GH, with albumin binding moiety attached (noncovalent albumin-binding properties)	7 days	Phase II studies in children and Phase III studies in adults
**GH-fusion proteins**			**Protein fused with GH**		
	Asterion	ProFuse GH	GH binding protein	1 month (planned)	Preclinical studies
	Genexine and Handok	GX-H9	Hybridization of noncytolytic immunoglobulin Fc portions of IgD and IgG4	7–14 days	Phase II studies in adults
	Hanmi Pharmaceutical Co.	LAPSrhGH/HM10560A	Homodimeric aglycosylated lgG4 Fc fragment	7–14 days	Phase II in adults
	OPKO Biologics and Pfizer	MOD-4023	Carboxyl-terminal peptide (CTP) of hCG β-Subunit	7 days	Phase II studies in children and Phase III studies in adults
	Teva	TV-1106	Albumin	7 days	Trials have been discontinued
	Versartis	VRS-317	XTEN sequence: naturally occurring hydrophilic amino acids	7–14 days	Phase III studies in children and Phase II studies in adults

In light of the interest generated by these new formulations, the Growth Hormone Research Society (GRS) convened a workshop in Asilomar, CA, USA on 4–7 November 2015. The purpose of the workshop was to review the current state of the field and address key issues regarding trial design, efficacy, and safety of LAGH preparations.

## Methods

Fifty-five invited international leaders from 14 countries across 5 continents with expertise in the field of GH attended the meeting. These included pediatric and adult endocrinologists, basic scientists, regulatory scientists from the FDA and EMA, and scientists from the pharmaceutical industry. A review of the current status of LAGH development was published before the meeting (9). A planning committee of the GRS comprising adult and pediatric endocrinologists from academic institutions determined the agenda, selected speakers to summarize key relevant topics, and formulated the questions for discussion at this workshop.

Following presentations that summarized the literature, four breakout groups addressed each topic in more detail by discussing the list of questions formulated by the planning committee and subsequently agreed upon by all participants. All attendees reconvened after each of the breakout sessions to share reports from the groups. At the end of days 1 and 2, a writing team compiled the breakout group reports into a final document that was reviewed and revised in its entirety by participants on the concluding day in an open forum. When there was no clear agreement by most participants, consensus was reached by voting. This draft document was edited further for formatting and references, and then recirculated to the academic attendees for final review after the meeting. Meeting participants from pharmaceutical companies were not part of the planning committee or writing team and were not present during the discussion and text revision on the final day. However, scientists from industry and regulatory agencies were shown the manuscript before submission in order to identify factual errors, but they were not involved in the writing. This report is a concise chronicle of the workshop and is not intended to be an exhaustive review of the literature on the topic. The contents of this manuscript were derived from: (i) the content of the speaker presentations, (ii) the combined comments of the breakout groups to the specific questions discussed, (iii) the collective remarks of the entire group during report-back sessions, and (iv) the current peer-reviewed literature in the GH field.

## The clinical need for a LAGH preparation in pediatric and adult endocrinology

Large interindividual variability in the growth response to daily GH injections in GH-deficient children and adults reflects numerous inherent biological factors, including dosing and dose frequency and varying sensitivity to both exogenous GH and endogenous insulin-like growth factor 1 (IGF1) (10, 11). In addition, reduced adherence to daily injections likely compromises response to treatment. The requirement for daily injections may also represent a barrier to initiating and maintaining therapy in both pediatric and adult patients. Many scenarios were discussed in which LAGH would represent a significant innovation in the delivery of clinical care for patients deficient in GH. All participants recognized the need for LAGH.

## Development of LAGH preparations

As described in the review prepared for this workshop (9), several technologies are being used to prolong GH exposure. These include: (i) depot formulations, (ii) pegylation, (iii) prodrugs, (iv) noncovalent albumin binding GH compound(s), and (v) GH-fusion proteins (Table 1). These preparations are currently in various stages of development, with some already approved in Europe and Asia.

## Endocrine features of LAGH

There was overall consensus that currently available data do not allow determination of whether the effects of LAGH will differ from those of daily GH. In addition, it is not clear whether the physiological GH pulsatility and diurnal secretory pattern seen in normal subjects are important for attaining optimal injected GH effects (12). It has been emphasized that many currently prescribed hormone replacement therapies, including daily GH, glucocorticoids, and various formulations of sex steroids, do not replicate normal physiologic pattern of secretion of the endogenous hormones in terms of pulsatility and diurnal variation. The LAGH preparations that were used in clinical trials, as well as continuous infusions of GH, did not result in tachyphylaxis or substantial differences in clinical effects when compared with daily injections of GH (13, 14, 15, 16).

## Safety considerations unique to LAGH

LAGH products exhibit different pharmacokinetics and pharmacodynamics compared with daily GH and, therefore, safety considerations may be specific to these formulations. In particular, the mechanism whereby each product prolongs the action of GH differs; such additional moieties or properties may have an impact on safety distinct from considerations about GH or IGF1 levels. Therefore, the safety of each individual LAGH product should be assessed. Also, there is a need to consider the pharmacodynamic impact of each LAGH product. Care should be taken to avoid overtreatment, and therefore safety monitoring is critically important. Potential issues include: (i) supraphysiological elevations of GH and/or IGF1, (ii) trough GH concentrations above normal physiological levels, (iii) fluctuating IGF1 levels, (iv) elevated IGF1 in the absence of GH bioactivity, (v) nonphysiological tissue distribution due to distinct biological features of the products, and (vi) the specific chemical composition of each LAGH product may confer unique safety issues.

The impact on safety of longer exposure to GH and/or IGF1 with the LAGH preparations is unclear. Serum IGF1 levels are generally higher immediately following an injection and lower before the next injection. It will be important to associate IGF1 levels with side effects.

In children and adolescents, rare side effects of daily GH replacement include intracranial hypertension, slipped capital femoral epiphyses, and hyperglycemia in those at risk for diabetes (17). Whether LAGH will exacerbate these risks is unknown. The safety issues need to take into account specific factors including age, pubertal stage, and populations at risk for diabetes (particularly certain GH therapeutic indications such as small for gestational age (SGA) and Turner syndrome). In patients who develop the rare and serious side effect of benign intracranial hypertension, daily GH therapy is suspended until the condition resolves. One theoretical (and important) concern with LAGH is that the long duration of action does not allow immediate reversibility of exogenous GH exposure. In young children with congenital, severe GH deficiency, hypoglycemia may occur when the drug effect wanes. In such patients, prospective studies would need to include monitoring blood glucose levels at the end of the LAGH dosing interval, when serum GH levels may be at their lowest.

In children and adults, it is unclear whether carbohydrate or lipid metabolism (18) will differ between LAGH and daily GH-treated patients. Glucose metabolism should be monitored in patients on LAGH because of concerns related to the possibility of prolonged GH bioactivity (19).

The theoretical concerns regarding whether there is an effect of GH treatment on neoplasia were addressed in recently published position papers (20, 21, 22). These concerns may also apply to LAGH. The impact of possible supraphysiological and sustained GH and IGF1 levels after an injection and the altered balance between GH and IGF1 with LAGH on potential long-term cancer risk is unknown. Childhood cancer survivors should be monitored for possible development of secondary neoplasms, especially those who received cranial irradiation.

Daily administration of GH results in a consistent day-to-day hormonal and metabolic response (23). In contrast, LAGH administration may result in different patterns on each day within the dosing interval, and the long-term consequences of this pattern are unknown. It is important to emphasize that neither daily GH nor LAGH products recapitulate normal physiologic secretion.

LAGH preparations may result in injection site pain, skin irritation, allergic reactions, and lipoatrophy (24). These effects, which were reported in some early LAGH studies, vary among preparations (25). The incidence of local skin reactions for each preparation should be explored further.

## IGF1 measurements during LAGH administration

Unlike the experience with daily GH, both the appropriate timing of blood sampling and the interpretation of the IGF1 standard deviation score (SDS) in LAGH-treated patients are controversial. LAGH preparations differ in the kinetics of serum GH and IGF1 that they induce. Studies need to take into account the pharmacokinetics and pharmacodynamics of each product in order to gauge the optimal timing of IGF1 measurement. The goal is to maintain serum IGF1 concentrations within the normal age-appropriate range for a majority of the treatment period.

## The role of antibody measurements

Antibody measurements are used to assess potential immunogenicity that may impact both efficacy and safety. Because the impact of these antibodies on clinical outcomes is unclear, the interpretation of such results and the assimilation into study design need to be considered. Specifically, it is clinically important to determine whether there are neutralizing antibodies that could inhibit the intended drug effects. Discontinuation of LAGH in the face of rising antibody titers may be considered if there is a clinically significant reduction in IGF1 response or growth velocity or coexistence of a severe allergic reaction. However, there are challenges in interpreting these results due to the marked variability of antibody assays.

## Pediatric trial design

The goals of pediatric GH therapy are to maximize adult height and to bring children into the normal height range as soon as possible. Primary outcomes include first year height velocity and delta height SDS (26); however, the most practical endpoint to measure in a Phase 3 pediatric clinical trial is height velocity. In addition, secondary outcomes include height velocity SDS, delta height velocity, bone age advancement, predicted adult height, and delta IGF1 SDS. Nongrowth outcomes should also be measured, including body composition, metabolic indices, and percentage of children entering puberty. Additional patient-related outcomes to be monitored include adherence, quality of life (QoL), and patient satisfaction. Models to assess burden of disease and therapy should be developed.

A trial duration of 1 year in prepubertal, treatment-naïve children was considered appropriate to assess efficacy using a carefully matched control group. Longer treatment duration is required to assess the safety profiles of the LAGH preparations. Weight-based fixed dosing was advised for pediatric Phase 3 trials, with downward dose titration performed for elevated serum IGF1 levels demonstrated on at least two consecutive measurements. It is important to establish the appropriate time between injections for IGF1 measurement. Opinions differ on the utility of peak, mean, and trough IGF1 concentrations as tools to monitor and guide therapy. Phase 2 results for each product should provide information to determine which IGF1 measurement time point is clinically useful.

It is important to ensure matched study populations when comparing LAGH with daily GH. Variables to consider should include age, gender, midparental target height, onset of puberty, ethnicity, severity of growth hormone deficiency (GHD), and BMI-SDS. It will be essential to define selective benefits and risks of LAGH in randomized controlled trials in pediatric populations other than GHD, such as Turner syndrome, chronic renal failure, Prader–Willi syndrome, Noonan syndrome, short children born SGA, and idiopathic short stature. It should not be assumed that the effects and safety profile of LAGH in these other conditions will be similar to those seen with LAGH therapy in GHD.

In clinical trials, safety monitoring should be performed after 1 and 3 months, and then every 3 months thereafter. This should include assessment of glucose regulation with hemoglobin A1c, fasting glucose, and insulin levels. Hepatic, thyroid, and adrenal function should also be monitored. However, in formal surveillance programs and clinical practice, these safety parameters should be considered annually.

## Adult trial design

Daily GH replacement therapy for adult GHD was approved by the United States FDA in 1996, using changes in body composition as the primary outcome. Additional outcome measures that could be evaluated in LAGH trials include patient-recorded outcomes such as QoL, exercise capacity, and work productivity. A trial duration of 6 months is considered appropriate to assess efficacy. As in the pediatric trials, it is important to include carefully matched control groups. Attention should also be paid to the many variables previously described including age, gender, severity of GHD, and BMI when comparing LAGH with daily GH therapy. Safety of LAGH in adults can only be determined after a long treatment duration, and therefore extension studies may be required after completion of the controlled trials.

Truncal or visceral fat reduction at 6 months is recommended as the primary end point in adult trials. Secondary end points (some of which would require longer treatment duration) include IGF1, QoL, lipid profiles, exercise capacity, cardiovascular risk factors, and bone mineral density. The initial dose should take into account age, gender, and oral estrogen use in women. Study design in adults should include a dose titration to achieve a mean serum IGF1 in the normal age-appropriate range, with the timing of the IGF1 measurement based on pharmacodynamic findings for each compound.

Safety monitoring with laboratory tests, similar to those described above for the pediatric trials, should be performed at 1, 3, and 6 months on treatment and then at regular intervals (sooner if clinically indicated) in clinical trial extensions, formal surveillance programs, and in clinical practice. Brain MRI should be performed before the start of LAGH and as clinically indicated thereafter in patients with GHD due to hypothalamic–pituitary tumors.

## Adherence

Adherence to therapy is an extremely important variable that is difficult to assess reliably. Clinical trials select for motivated participants and the infrastructure of these studies enhance adherence. Methods to monitor adherence include vial count, electronic devices, web-based entry, and patient diaries. Serum IGF1 may be a surrogate marker of compliance. Measurement of LAGH by product-specific assays may be useful. Adherence may also be affected by ease of use of the delivery device, but little data are available to assess this supposition (27, 28).

## Conclusion

LAGH compounds are a novel approach to the therapy of GHD and other growth disorders. LAGH may represent an advance over daily GH injections because of the potential for improved adherence and outcomes. LAGH may offer patients and families therapeutic alternatives and flexibility. Better methods to assess adherence need to be developed and validated. Long-term surveillance registries that include assessment of long-term efficacy, cost-effectiveness, disease burden, QoL, and safety measures are essential for understanding the impact of chronic exposure to all preparations of LAGH.

## Declaration of interest

J S C: consultant for Ascendis Pharma A/S, Novo Nordisk, and Teva. P F B: consulted for Ipsen, Novo Nordisk, EMD Serono, Versartis, and Sandoz, and currently receives grant support from Versartis. M B: consults for Genexine, OKPO, Sandoz, Teva, and Versartis, and has previously consulted for Biopartners. B M K B: PI, research grants to Massachusetts General Hospital from OPKO Biologics, Novo Nordisk, Versartis. Consultant to: Novo Nordisk, Pfizer, and Versartis. M C S B: consulted for Pfizer; lecture fees from Pfizer, Merck Serono. F F C: research grants, lecture, or consulting fees from Pfizer. PhC: research and educational grants from Ipsen, Novo Nordisk, and has served as investigator for clinical trials funded by Pfizer, Ipsen, Prolor Biotech. Member of Advisory Boards from Ipsen, Eli Lilly. Gave lectures for Ipsen and Pfizer. All the fees and honoraria were paid to his institution. PierreC: consulted for, principal investigator of a clinical trial, and speaker or chair in meetings promoted by Merck Serono and Ascendis Pharma A/S. C S C: research support from Merck Serono. D R C: consulted for Pfizer and Novo Nordisk. L E C: honorarium from Scherer Clinical Communications. PinchasC: consults for Ascendis Pharma A/S, OPKO Biologics, Teva, and Versartis. J F: nothing to disclose. A G: consults for the Pfizer International Growth Study database. Y H: nothing to disclose. M W H: nothing to disclose. K H: receives lecture fees from Ipsen and consults for Versartis and Pfizer. A R H: consults for Teva, Ascendis Pharma A/S, Versartis, and Genexine. J H: nothing to disclose. R H: nothing to disclose. C H: investigator for Novo Nordisk, Teva, Ascendis Pharma A/S, Versatis, and Handok-Genexine and has consulted for Novo Nordisk and Teva. J O L J: lecture fees and grant support from Pfizer and Novo Nordisk. G J: lecture fees from Novo Nordisk, Pfizer, and Merck Serono and has received grant support (2010–2015) from Novo Nordisk, Pfizer, and Sandoz. A J: lecture fees from Ipsen, Novo Nordisk, Ferring, and Sandoz; principal investigator on a multicenter trial, which received unrestricted grant support from Novo Nordisk. L K: nothing to disclose. J J K: consults for Teva and EMD Serono; lecture fees from Pfizer; inventor of Growth Hormone Antagonists (US Patent # 5,350,836). K O L: nothing to disclose. K W L: nothing to disclose. X L: consults for Gensci, Ipsen, Merck Serono, Novo Nordisk. S M: research grant support from Versartis. B S M: consults for Alexion, BioMarin, Endo Pharmaceuticals, Genentech, Ipsen, Novo Nordisk, Sandoz, and Versartis and has received research support from Alexion, Abbvie, Eli Lilly, Debiopharm, Endo Pharmaceuticals, Genentech, Ipsen, Novo Nordisk, Pfizer, Sandoz, and Versartis. M M: consulted for Genentech. V P: PI in clinical research studies for LG Life Science, OPKO Biologics, Versartis, Hanmi, and Teva. R G R: consults for Ascendis Pharma A/S, OPKO Biologics, Versartis, Teva, Genexine, Novo Nordisk, and Sandoz. J R: research support from Eli Lilly, Novo Nordisk, and Versartis and consults for and serves on an advisory board for Novo Nordisk. R J R: equity interests in Diurnal Ltd and Asterion Ltd. P S: consults for Ascendis Pharma A/S, Teva, OPKO Biologics. Speaker bureau Novo Nordisk. C J S: consults for Prolor, Versartis, Novo Nordisk, Merck Serono, Pfizer, and Genexine. M O T: Founder and CSO Ammonett Pharma, which is developing a ghrelin mimetic. H W: nothing to disclose. K Y: research grant support from Pfizer, Novo Nordisk, Eli Lilly, OPKO Biologics, Teva, and Versartis, and has served on the advisory boards for Pfizer, Novo Nordisk, Sandoz, and Versartis.

## Funding

This work was supported by the Growth Hormone Research Society.

## Dedication

The authors dedicate this paper to our late colleague and friend, Jens Sandahl Christiansen, a consummate clinical investigator and a kind and devoted physician.
